# Functional impact of glycogen degradation on astrocytic signalling

**DOI:** 10.1042/BST20140157

**Published:** 2014-09-18

**Authors:** Margit S. Müller

**Affiliations:** *Department of Pharmacology, University of Cambridge, Tennis Court Road, Cambridge CB2 1PD, U.K.

**Keywords:** astrocyte, ATP, energy metabolism, glycogen, glycogen phosphorylase, signalling, AC, adenylate cyclase, AR, adrenergic receptor, ER, endoplasmic reticulum, Gln, glutamine, Glu, glutamate, GP, glycogen phosphorylase, IP_3_, inositol 1,4,5-trisphosphate, NA, noradrenaline, NKA, Na^+^/K^+^-ATPase, NKCC1, Na^+^–K^+^–Cl^−^ co-transporter 1, PK, phosphorylase kinase, PKA, protein kinase A, sAC, soluble adenylate cyclase, SERCA, sarcoplasmic/endoplasmic reticulum Ca^2+^-ATPase, SOCE, store-operated calcium entry, STIM1, stromal interacting molecule 1, TRP, transient receptor potential

## Abstract

Astrocytic glycogen degradation is an important factor in metabolic support of brain function, particularly during increased neuronal firing. In this context, glycogen is commonly thought of as a source for the provision of energy substrates, such as lactate, to neurons. However, the signalling pathways eliciting glycogen degradation inside astrocytes are themselves energy-demanding processes, a fact that has been emphasized in recent studies, demonstrating dependence of these signalling mechanisms on glycogenolytic ATP.

## Introduction

Glycogen, the storage form of glucose in animals and fungi, provides cells with a readily accessible pool of metabolic energy. The complex polysaccharide was first isolated and described in the 19th Century by Bernard [1]. In his studies, Bernard also recognized the associated enzymatic activity, noting that glycogen degradation persists post-mortem.

As early as 1972, it was reported that brain glycogen, residing almost exclusively in astrocytes [2], appears to be coupled with neuronal activation, with levels increasing during anaesthesia [3]. Today, brain glycogen is generally accepted as a dynamic player in brain energy metabolism, particularly during enhanced brain activation in the context of functions such as learning and memory.

Astrocytic glycogen degradation is often regarded only in the light of either providing lactate as an energy substrate to neurons as part of the astrocyte–neuron lactate shuttle hypothesis [4,5] or sparing blood-borne glucose for use by neurons [6]. However, it is clear that no cellular signalling mechanism is energy-neutral: on some level, every cellular process is dependent on provision of ATP. Consequently, the signalling cascades triggering astrocytic glycogen degradation are energy-requiring mechanisms and it is plausible to assume that some of the glycogenolytic ATP generated by these pathways is used for their own energetic support. Indeed, recent studies have indicated that glycogen-mobilizing signal transduction pathways are impaired upon inhibition of glycogen degradation [7,8]. Such a function is reflected by the sensitivity of the glycogen-degrading enzyme glycogen phosphorylase (GP) to increases in cytosolic AMP, generated mainly by the activity of adenylate kinase (AK), which, upon hydrolysis of ATP to ADP, catalyses interconversion of two ADP into one ATP and one AMP.

The present review focuses on the most prominent glycogen-degrading signals in astrocytes, highlighting their metabolic demands and evidence for reliance on glycogenolytic ATP.

## Glycogen phosphorylase

Degradation and synthesis of glycogen are regulated in opposing ways, with phosphorylation and increased AMP concentration activating degradation and at the same time inhibiting synthesis [9]. GP, the key enzyme in glycogen degradation, occupies a prominent position in the history of biochemistry, as studies of GP led to the discovery of the two most important mechanisms in enzymatic regulation.

GP was first characterized in the 1930s by Cori et al. [10]. A few years later, the same group demonstrated that GP can be activated by AMP, a study that constitutes the discovery of allosteric regulation [11]. Building on this work, Krebs and Fischer [12], also working on GP, discovered the regulation of enzymes by reversible phosphorylation.

**Figure 1:**
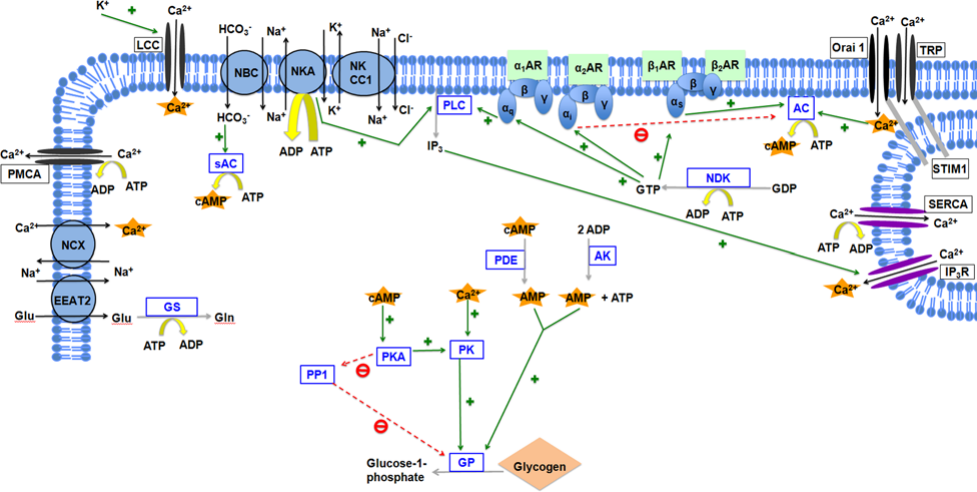
Schematic diagram of glycogenolytic stimuli in astrocytes GP is regulated by phosphorylation in a cAMP- and Ca^+^-dependent manner, and activated allosterically by AMP. Four major glycogenolytic stimuli, K^+^ uptake, Glu uptake and conversion into Gln, NA and SOCE are depicted with their associated signalling pathways. Orange stars emphasize sources of the GP stimuli cAMP, Ca^2+^ and AMP. Yellow rounded block arrows symbolize sites of ATP hydrolysis. Black arrows represent transport of ions; grey arrows show enzymatic reactions, with enzymes depicted in blue squares. Green arrows, accompanied by a green plus, symbolize activation, whereas red broken arrows with a minus show inhibition. For details, see the text.

Accordingly, GP is activated allostericaly by AMP and also covalently via phosphorylation, the latter resulting from a signalling cascade that can be triggered by either Ca^2+^ or cAMP [13]. Increase in cytosolic Ca^2+^ directly activates the GP-phosphorylating enzyme phosphorylase kinase (PK) by binding to its calmodulin subunit. PK is also activated by protein kinase A (PKA), which in turn is stimulated by cAMP (Figure 1). Both Ca^2+^ and cAMP-dependent phosphorylation are necessary for PK to be fully activated [14].

The relative importance of cAMP and Ca^2+^ in activation of astrocytic GP has been debated in the literature, with either of the two messengers being ascribed the prominent role, depending on the outcome of the respective study [9]. In particular, it has been pointed out that, at least in muscle, Ca^2+^ binding is an absolute requirement for PK activity [15]. However, in this context, it is important to consider that, in addition to promoting phosphorylation of GP by PK, cAMP via PKA also leads to inhibition of the enzyme dephosphorylating GP, protein phosphatase 1 (PP1), further shifting the equilibrium towards activation of GP [16]. Therefore, even if Ca^2+^ binding should be a prerequisite for substantial activation of PK, increase in cAMP alone can be expected to lead to a disinhibition of GP, shifting the balance towards glycogen degradation (Figure 1).

Moreover, cytosolic cAMP concentration is tightly coupled to Ca^2+^ signalling. Depending on the respective isoform, both adenylate cyclase (AC), the enzyme generating cAMP, and phosphodiesterase (PDE), which catalyses cAMP degradation, can be positively or negatively regulated by Ca^2+^ [17–19].

## Noradrenergic signalling

Although the somata of noradrenergic neurons are confined to two brain stem structures (locus coeruleus and lateral tegmental field), their projections reach throughout the entire cortex and cerebellum, making noradrenaline (NA) an important and versatile modulator in various processes, such as circadian control or learning and memory [20,21]. Moreover, adrenergic signalling in the central nervous system (CNS) is not restricted to neurons, as astrocytes have been shown to express α_1_-, α_2_-, β_1_- and β_2_-adrenergic receptors (ARs) [22]. In fact, NA was the first glycogenolytic transmitter to be described for the brain [23].

Adrenergic signalling can exert opposing influences on astrocytic glycogen metabolism: it can either stimulate glycogen degradation by increasing cytosolic Ca^2+^ or cAMP, or inhibit degradation by inhibiting cAMP production (Figure 1). Indeed, it has been reported that prolonged adrenergic stimulation of astrocytes leads to a biphasic glycogen response, with an initial substantial glycogen degradation, followed by glycogen resynthesis [24,25]. Although the initial glycogenolytic effect of NA on astrocytes is very robust, the specific signalling cascades and relative contribution of the different adrenoreceptor subtypes are debated, with conflicting evidence appearing to result from a dependence on species, developmental stage and experimental preparation [9,25–27]. It has indeed been shown that the glycogenolytic response of astrocytes to NA changes throughout development [28]. As the nature of these conflicting reports is beyond the scope of the present review, I focus on some of the signalling cascades most prominently associated with each of the adrenoreceptors in astrocytic glycogen metabolism.

Both β_1_-AR, and β_2_-AR are G_s_-coupled receptors: agonist-binding leads to a conformational change in the receptor and a dissociation of the α-subunit, which subsequently binds to AC, stimulating production of cAMP [29,30]. α_1_-AR, in contrast, is a G_q_-coupled receptor, activating phospholipase C (PLC), which catalyses hydrolysis of phosphatidylinositol 4,5-bisphosphate, generating diacylglycerol and inositol 1,4,5-trisphosphate (IP_3_), the latter triggering release of Ca^2+^ from endoplasmic reticulum (ER) stores [31,32].

Interestingly, astrocytes can also express α_2_-AR, which as a G_i_-coupled receptor would be able to inhibit cAMP generation and therefore suppress glycogen degradation and stimulate glycogen synthesis, implying that the same messenger could induce mobilization and replenishment of astrocytic glycogen stores. This concept has been experimentally verified, showing that NA acting via α_2_-AR can increase astrocytic glycogen turnover [26].

There are several ATP-requiring steps involved in adrenergic signalling, the most immediate being recovery of GTP. As G-protein-coupled receptors, ARs rely on provision of GTP upon ligand binding and signalling is terminated by hydrolysis of GTP to GDP. To regain GTP for new receptor activation, nucleoside diphosphate kinase (NDK) catalyses transfer of the γ-phosphate from ATP to GDP [33], therefore hydrolysing one molecule of ATP per receptor activation. Further downstream of the receptors, ATP is required for formation on cAMP. Finally, Ca^2+^ released from the ER upon IP_3_ signalling has to be removed from the cytosol by the actions of Ca^2+^-ATPases, such as plasma membrane Ca^2+^-ATPase (PMCA) and sarcoplasmic/endoplasmic reticulum Ca^2+^-ATPase (SERCA).

## Potassium uptake

Already 25 years ago, it was discovered that increased extracellular K^+^, released during neuronal excitation, is taken up by astrocytes, triggering glycogen degradation [34]. Evidence suggests that astrocytic K^+^ uptake is mainly facilitated by the Na^+^/K^+^-ATPase (NKA), and at very high extracellular K^+^ concentration also by the Na^+^–K^+^–Cl^−^ co-transporter 1 (NKCC1) [35,36]. NKCC1 is no longer expressed in neurons after development, and astrocytic NKA has lower affinity and higher capacity than neuronal NKA, rendering astrocytic uptake of excess extracellular K^+^ a highly efficient process [37,38].

K^+^ uptake can influence an array of ion exchangers and signalling pathways, downstream of which either Ca^2+^ or cAMP can be increased, activating glycogen degradation (Figure 1).

### Ca^2+^

Two distinct sources of cytosolic Ca^2+^ increase have been shown to be involved in K^+^-stimulated glycogen degradation: opening of L-type Ca^2+^-channels and NKA-mediated activation of IP_3_ signalling [7]).

### cAMP

Astrocytes have recently been shown to express a bicarbonate (HCO_3_^−^)-sensitive soluble adenylate cyclase (sAC) [39]. sAC is activated indirectly during astrocytic K^+^ uptake, as Na^+^ extruded by increased NKA activity is taken back up through the Na^+^–HCO_3_^−^ co-transporter (NBC), increasing cytosolic HCO_3_^−^. This study has demonstrated that cAMP generated by sAC, following this pathway, directly activates glycogen degradation.

As NKA is the main facilitator of astrocytic K^+^ uptake, it is evident that the process is heavily dependent on ATP supply. Furthermore, as in the case of NA signalling, cAMP generation and Ca^2+^ homoeostasis require additional ATP. Recently, it has been demonstrated that astrocytic K^+^ uptake is specifically dependent on ATP provided by glycogenolysis, as inhibition of GP abolished uptake, even in the presence of extracellular glucose [7,39].

## Glutamate uptake

One of the key functions of astrocytes in supporting neurotransmission is rapid uptake of glutamate (Glu) for swift termination of excitatory signalling. Glu is taken up in a sodium-dependent manner through the excitatory amino acid transporter 2 (EAAT2), following the sodium gradient created by activity of the NKA. Upon uptake, glutamine (Gln) synthetase catalyses ATP-dependent condensation of Glu and ammonia to Gln, which subsequently is released and taken up by neurons to be converted back into Glu [40]. This Glu–Gln cycle is important not only for termination of excitatory signals, but also for replenishing neuronal Glu content, as neurons do not express pyruvate carboxylase and are therefore unable to sustain their neurotransmitter pool by net synthesis of Glu. To sustain this cycle, astrocytes require two molecules of ATP per molecule of recycled Glu: one molecule of ATP for fuelling NKA and one molecule for Gln synthetase.

Studies have demonstrated that uptake and recycling of Glu are glycogen-dependent, even in the presence of extracellular glucose [41]. It has been suggested that this requirement for glycogen is due to a rapid increase in demand for ATP, in conditions of intense neuronal firing [42]: to sustain this acute energy demand, the rate of glycolysis has to increase, relying on glycogen degradation to supplement glucose 6-phosphate for glycolysis. Simultaneously, the co-transport of Na^+^ ions can activate the reverse mode of the sodium/calcium exchanger (NCX), leading to an influx of Ca^2+^ and activating glycogen degradation [43] (Figure 1).

In addition to providing ATP for the Glu–Gln cycle, glycogen has also been shown to be a preferred source of the carbon skeleton for astrocytic synthesis of Glu, a process suggested to be involved in memory consolidation [44].

## Store-operated Ca^2+^ entry (SOCE)

The functional significance of the ER as a dynamic Ca^2+^ store necessitates robust mechanisms maintaining its high Ca^2+^ content. Accordingly, SOCE and its associated proteins are found ubiquitously across cell types, including astrocytes [45]. Stromal interacting molecule 1 (STIM1), a transmembrane protein contained in ER membranes, is now widely accepted as the Ca^2+^-sensing component of the SOCE machinery [46], whereas at least two families of proteins mediate the associated entry of Ca^2+^ across the plasma membrane: transient receptor potential (TRP) channels [47] and Orai proteins [48]. STIM1 contains two Ca^2+^-binding EF-hand domains on its luminal side. Upon a decrease in Ca^2+^ concentrations inside the ER lumen, Ca^2+^ dissociates from STIM1, resulting in a conformational change of STIM1 that facilitates its accumulation in oligomers. These STIM1 aggregates migrate inside the ER membrane, towards sites close to SOCE channels in the plasma membrane, where they form discrete puncta [49]. Following STIM1 puncta formation, either TRP channels or Orai pores open, allowing influx of Ca^2+^ from the extracellular space.

Upon entry of Ca^2+^ through SOCE channels, the final step of SOCE is catalysed by SERCA, which pumps the Ca^2+^ ions into the ER, thus refilling stores [50].

Currently, there is no consensus about the molecular identity of the SOCE-mediating proteins in astrocytes, as conflicting results show functional expression of either Orai or TRP channels [45]. Thus it has to be assumed that either of these proteins can be responsible for observed astrocytic SOCE, depending on species and experimental model.

Recently, we have shown that Ca^2+^ entry through SOCE channels robustly stimulates glycogenolysis in both cerebellar and cortical murine astrocytes [8]. This stimulation is, at least in part, mediated by activation of AC and production of cAMP. Furthermore, as SERCA function is ATP-dependent, we investigated the possible dependence on glycogenolytic ATP. Indeed, inhibition of GP appeared to decrease ER calcium loading, indicating that a functional consequence of SOCE-triggered glycogenolysis in astrocytes is provision of ATP to energetically support SERCA function (Figure 1).

## Conclusion

Insight into the functional consequences of biochemical mechanisms requires an understanding of the regulatory pathways and dynamics that underlie them. The critical role occupied by glycogen in metabolic support of brain function is far from being completely understood. However, recent work has provided evidence that, in addition to the commonly discussed function of providing or sparing energy substrates for neurons, astrocytic glycogenolysis contributes to the ATP pool of astrocytes themselves, energetically supporting astrocytic signal transduction [7,8].

As no biological process is energy-neutral, any signalling pathway at some point will be dependent on provision of ATP. Therefore any signal leading to phosphorylation of GP will, at some point, be using ATP, producing AMP. Consequently, without careful experimental probing, there can be no clear distinction between the contribution of allosteric modification through AMP, and phosphorylation triggered by Ca^2+^ and cAMP to GP activation upon a given stimulus.
